# Retention of Fractural Maxillæ

**Published:** 1877-10

**Authors:** 


Retention of Fractured Maxillæ.—Fractures of the inferior maxilla are far more frequent than those of the upper maxilla. Some of these fractures are right difficult to reduce and retain in position, while in many cases the deformity is slight, and retention in position is easily accomplished. Hence, a double cause for the number and variety of appliances used in practice. Some are quite ingenious, and difficult of application; others are very simple, and easily applied.

In two cases of fracture—the first, fracture of the superior maxilla, just released from treatment, the second, of the inferior maxilla, yet under observation—india rubber rings, made of narrow sections of rubber tubes, and pased over the neighboring teeth, served the purpose of retention admirably well.

The first occurred in a French creole, twenty-six years of age, who, in an unsolicited bull fight, was knocked down, and though a fallen foe, was stamped in the mouth by his cowardly adversary. The right superior maxilla was fractured. A section of bone about one and a half inches square, including the alveolar process in front and part of the hard palate, was so displaced as to make an ugly deformity.

The piece of bone was adjusted and retained in place by a couple of rubber rings for twenty-five days, when the union was complete.

The second case is that of an Irishman, thirty years of age, who entered a surgical ward in the Charity Hospital, on the 16th of August 1877, with a double fracture of the inferior maxilla, caused by a blow with a brick-bat. One fracture occurred about an inch to the right of the symphysis, the other just in front of the left angle, both compound and comminuted. The tendency to displacement was hard to overcome, the application of an external splint, with a bandage closely applied, and the suturing of the teeth with the largest size silver wire, proving inadequate.

Graduated rubber rings, made of sections of tubes of different sizes, were passed over the first, second and third teeth from the seat of fracture, which held the loose section of bone firmly in place. A pasteboard splint, with a four-tailed bandage, was also applied. This dressing was made one week after the injury. One month after the injury, union was still incomplete, and fragments of necrosed bone were removed. Perhaps a more artistic appliance to such a fracture would not have secured a better result. Even in this exceptionally bad case, the efficiency of the rings was clearly shown.

The elasticity of the rubber rings is sufficiently powerful to overcome any muscular contraction which might tend to disarrange the parts, and constantly keeps the fractured edges of bone in close approximation. Indeed, a force too strong may displace the teeth, especially those next the seat of fracture.

Now, we do not urge the substitution of these rubber rings, in all cases of fracture of the maxillary bones, for the more admirable contrivances which we have at our command, such as the interdental splint of vulcanized rubber. We are very often forced to utilize what we have, in the absence of what we need. A great many fractures, even of the lower maxilla, are very easily held in proper position, and we would only suggest the use of these rubber rings as a cheap and simple device, the value of which we hope will recompense for the space occupied by this article.—*New Orleans Medical and Surgical Journal.*

